# ESCRTing proteasomes to the lysosome

**DOI:** 10.1371/journal.pgen.1008631

**Published:** 2020-03-19

**Authors:** Nava Segev

**Affiliations:** Department of Biochemistry and Molecular Genetics, College of Medicine, University of Illinois at Chicago, Chicago, Illinois, United States of America; Ohio State University, UNITED STATES

## Cellular recycling: Proteasome versus lysosome

Clearance of excess or damaged cellular components is crucial for homeostasis and under stress and defects in this process result in multiple diseases, including neurodegeneration [1]. This clearance is done in two sites: the proteasome, which degrades proteins, and the lysosome, which can degrade all macromolecules and organelles. Proteasomes are multi-protein machines composed of a 20S core particle (CP) capped with two regulatory particles (RP), which direct ubiquitinated proteins to the core cavity, where proteases reside [2]. The lysosome, a membrane-bound organelle with an acidic environment, contains diverse degradative enzymes that function in this milieu. In addition to endocytosis, in which endosomes fuse with lysosomes, two other ways lead into the lysosome: macro-autophagy and micro-autophagy. In macro-autophagy, the double-membrane autophagosome (AP) engulfs cargo and fuses with the lysosome, whereas in micro-autophagy cargo is engulfed by the lysosomal membrane itself. While there is plenty of information about macro-autophagy, not much is known about micro-autophagy [3]. In both cases, the membrane engulfing the cargo needs to seal and the mechanism of this sealing has been a major question in the autophagy field.

**Fig 1:**
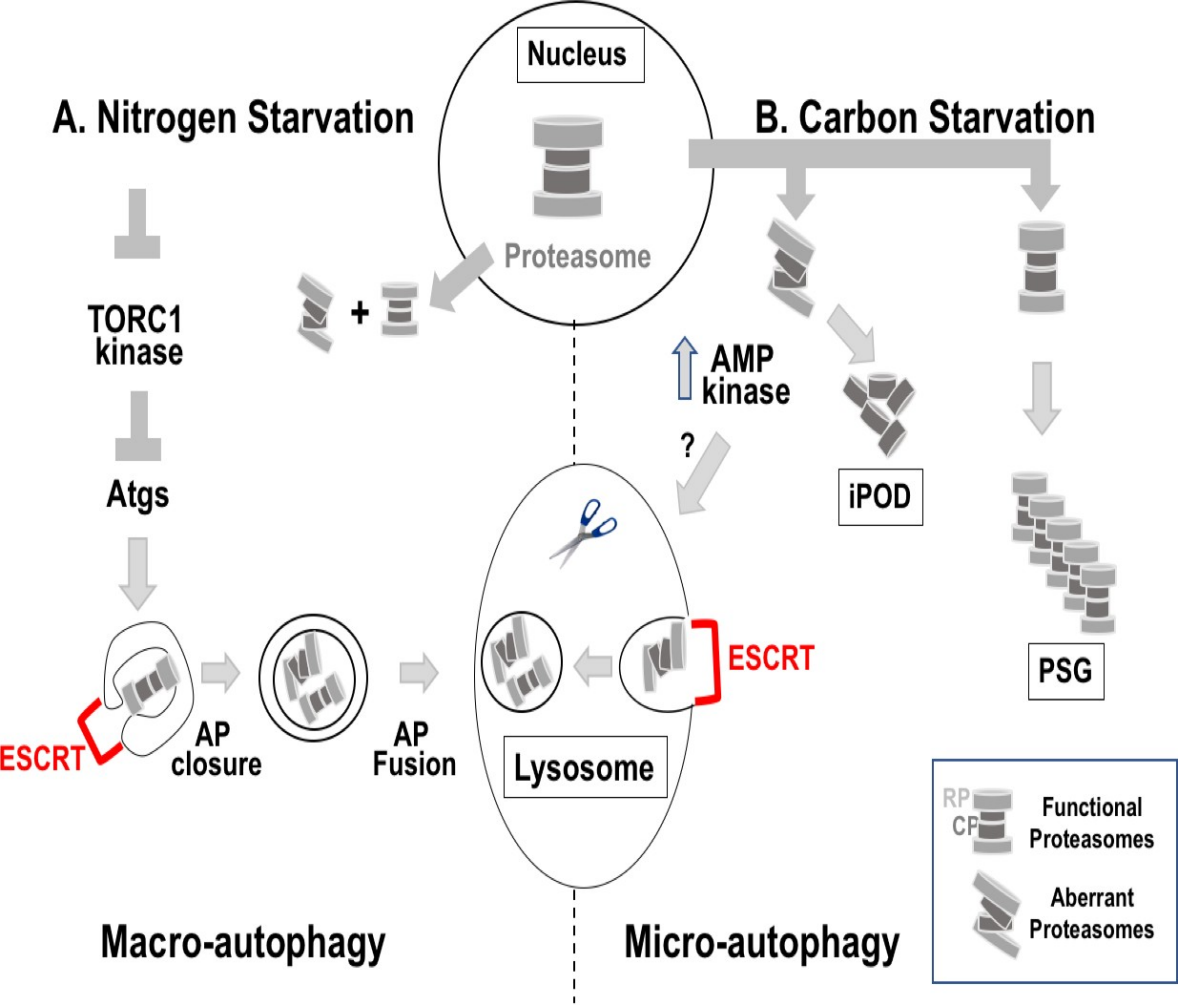
Proteasome fate during starvation in yeast. During normal growth, proteasomes are enriched in the nucleus. Upon starvation, proteasomes relocate to the cytoplasm. Under nitrogen starvation (A, left), TORC1 kinase inhibition stimulates macro-autophagy. In this pathway, Atgs form the double-membrane autophagosomes (APs), which engulf cellular components, including functional and damaged proteasomes, and fuse with the lysosome, a degradative compartment. Under glucose starvation (B, right), functional proteasomes are stored in proteasome storage granules (PSG). The fate of aberrant proteasomes depends on AMP kinase. AMPK induces micro-autophagy in which proteasomes are engulfed by the lysosomal membrane itself in a process termed micro-autophagy. Sealing of APs in macro-autophagy (A) and the lysosomal invagination in micro-autophagy (B), is mediated by the ESCRT complex. In the absence of AMPK, the core particles (CP) of proteasomes accumulates in the insoluble protein deposit (iPOD). See Table 1 and text for more details.

Another question in the degradation field has been how proteasomes recycle. During normal growth of yeast cells, the majority of the proteasomes reside in the nucleus and damaged proteasomes are shuttled to the lysosome via a selective macro-autophagy pathway, proteaphagy [2]. During stress, proteasomes relocate to the cytoplasm. Under nitrogen starvation, macro-autophagy is vastly induced and excess of cellular components, including proteasomes, are delivered to the lysosome (vacuole in yeast) for degradation. It was recently shown that in nitrogen-starvation induced macro-autophagy, ESCRT complexes play a role in sealing APs to allow their fusion with the lysosome [4]. Under glucose starvation, normal proteasomes are stored in proteasome storage granules (PSGs), which dissolve when glucose is added back [2]. However, it was not clear what happens to aberrant proteasomes under carbon stress (Fig 1).

Li et al., address this question [5]. They identified the AMPK and ESCRT complexes (see Table 1) in a genome-wide screen for gene-deletion mutants defective in proteasome degradation during glucose starvation and determined their effect on proteasome fate. Their three major findings are: First, under carbon stress, damaged proteasomes are delivered to the lysosome via micro-autophagy in an AMPK- and ESCRT-dependent manner. Second, when AMPK is absent, the CP of proteasomes accumulate in the iPOD, a deposit site for protein aggregates [6]. Third, whereas under nitrogen starvation proteasomes are degraded together with other cellular components, under carbon starvation proteasomes are sorted to different destinations and only damaged proteasomes are degraded (Fig 1). This sorting is an example of a more economical solution of cells to carbon stress than the extensive degradation of cellular components that occurs under nitrogen starvation.

**Table 1:** Glossary. Players associated with this perspective and their description.

Player	Description
**Recycling Compartments**	
**Proteasome**	Protein complex that degrades ubiquitinated proteins. Found in the nucleus and cytoplasm Composed of core (CP) and regulatory (RP) particles
**Lysosome**	A cytoplasmic organelle (vacuole in yeast) with a low PH. Degradative enzymes surrounded by a single membrane
**Autophagosome**	AP: a double-membrane cytoplasmic organelle. Composed of autophagy related proteins (Atgs) and membrane. Engulfs cargo and fuses with the lysosome
**Storage Compartments**	
**PSG**	Proteasome storage granules. Reversible storage site for functional proteasomes
**iPOD**	Insoluble protein deposit: a major deposition site of protein aggregates. Locates near the lysosome and AP formation site. Without AMPK, CPs of aberrant proteasome are deposited in iPOD
**Kinases**	
**TORC1**	Protein kinase—target of rapamycin complex 1. Positive regulator of cell growth; negative regulator of macro-autophagy. Under nitrogen starvation: TORC1 is inhibited, macro-autophagy is induced
**AMPK**	5' adenosine monophosphate-activated protein kinase. Activated under when ATP levels are low, including under low glucose. AMPK signaling coordinates multiple cell responses to low ATP
**Autophagy Pathways**	
**Macro-autophagy**	Cargo delivery to lysosome requires Atgs and APs
**Micro-autophagy**	Cargo delivery to lysosome does not require ATGs and APs
**Membrane Sealer**	
**ESCRT**	Endosomal sorting complexes required for transport. Four ESCRT 0-III complexes plus accessory factors, e.g., Vps4. Seal membranes (by scission from a neck) in multiple processes

The emerging picture puts the lysosome at the top of the cell food chain: While both proteasomes and the lysosome can degrade cellular components, proteasomes get degraded by the lysosome [2]. Interestingly, depending on the stress, proteasomes reach the lysosome in different ways, about which multiple questions are still open.

## Proteasome locations

When proteasomes move to the cytoplasm under stress, they localize to three locations depending on the type of stress: PGSs, iPOD or the lysosome. The two major fates of normal (un-damaged) proteasomes are reversible storage in PSGs during carbon starvation, and degradation in the lysosome during nitrogen starvation. Non-functional proteasomes are degraded in the lysosome during both kinds of stress. Under nitrogen starvation, proteasomes are delivered to the lysosome via macro-autophagy. However, under glucose starvation, macro-autophagy is not stimulated, and damaged proteasomes reach the lysosome via AMPK-dependent micro-autophagy.

iPOD, a major deposition site of protein aggregates, was previously thought to be a stop for damaged proteasomes *en route* to the lysosome [6]. However, Li et al., show that during carbon stress exit of damaged CPs from the iPOD is dependent on AMPK. In the absence of AMPK, delivery of proteasomes to iPOD is a dead-end road [5]. One remaining question is whether this route also applicable to other protein aggregates that accumulate in iPOD.

## TORC1 versus AMPK–upstream regulation

Two conserved kinase complexes regulate multiple signaling pathways during cellular stress, including autophagy. During normal growth, the TORC1 kinase complex promotes cell growth and proliferation while inhibiting macro-autophagy. In yeast, under nitrogen starvation, TORC1 is inhibited and the macro-autophagy pathway is activated. AMPK is activated when intracellular ATP levels are low, e.g., under glucose starvation. These two major sensors of nutrients and energy, TORC1 and AMPK, respectively, can inhibit each other directly or indirectly [7]. However, because they are mostly activated under different conditions, it is not clear how their antagonistic signaling plays out especially with regard to autophagy.

While Li et al., follow the fate of proteasomes during carbon starvation, the implication of their results is that AMPK does not promote macro-autophagy during carbon stress. Instead, it promotes delivery of damaged proteasomes to the lysosome via micro-autophagy. Future research should define mechanisms underlying such a selective process. Regardless, this implies that AMPK promotes a more economical recycling of cellular components than the massive degradation induced during nitrogen starvation due to TORC1 inactivation.

## Macro- versus micro-autophagy

Currently, very little is known about the regulation and mechanisms specific for micro-autophagy. Micro-autophagy can be induced in yeast by carbon, endoplasmic reticulum (ER) or nitrogen stress. Li et al., report that carbon depletion causes micro-autophagy of aberrant proteasomes in an AMPK-dependent manner [5]. ER stress results in micro-autophagy of the ER in a Nem1/Spo7-dependent way [8]. Finally, micro-autophagy can also be induced by TORC1 inactivation under nitrogen starvation in a Nem1/Spo7-dependnt way [9]. However, neither AMPK nor the Nem1/Spo7 phosphatase complex, which regulates membrane domains, specifically promote micro-autophagy and both were implicated also in macro-autophagy [9, 10]. Thus, it seems that micro-autophagy can be induced by multiple stresses through multiple signaling pathways, and further research is needed to identify its specific upstream regulators.

The same is true for machinery components specific for micro-autophagy. While, macro-autophagy is dependent on Atgs that are required for the formation of APs, neither play a role in micro-autophagy [3]. The three aforementioned studies show that micro-autophagy is dependent on subunits of the ESCRT complex [5, 8, 9]. However, ESCRT is also required for macro-autophagy [4]. Future research is needed to shed light on what triggers the lysosomal membrane to engulf certain cellular components and the mechanisms of this process downstream of the AMPK signaling (question mark in Fig 1B).

## ESCRT in autophagy

ESCRT is required for sealing AP membrane in macro-autophagy [4] and lysosomal membrane in micro-autophagy of proteasomes, ER and other cargos [5, 8, 9]. Thus, the repertoire of the four ESCRT complexes together with the Vps4 ATPase expends from endosome maturation, cytokinesis and viral release [11] to both autophagy types.

How does ESCRT function in autophagy? In endosomes, ubiquitinated cargos are sorted by ESCRT-0 to membrane subdomains and ESCRT complexes I-III assemble to promote membrane sealing by the Vps4 ATPase [11]. In macro- and micro-autophagy of proteasomes, mutations in subunits representative of all the ESCRT complexes result in defects, but the Vps4 ATPase seems to not be required for the latter [4, 5]. In micro-autophagy of ER, a role for subunits of ESCRT-I and ESCRT-III were tested [8] while in micro-autophagy under nitrogen starvation only a subunit of ESCRT-0 was tested [9]. Thus, a more thorough analysis of ESCRT complexes is needed for understanding how ESCRT gets to the AP and lysosomal membranes and how it functions in macro- and micro-autophagy. In addition to sealing membranes, ESCRT might play a role in cargo sorting in autophagy.

## Conservation

Li et al., report the recycling of proteasomes via an AMPK-dependent micro-autophagy, accumulation of defective proteasomes in iPOD and a role of ESCRT in autophagy in yeast [5]. A looming question is whether these findings are conserved in human cells. All the players (Table 1), including TORC1 and AMPK, are conserved from yeast to human cells [7], aggresome is the human equivalent of iPOD [12], and stress-induced autophagy of proteasomes [13] and a role for ESCRT-III in AP sealing were reported in human cells [14]. Therefore, principle mechanisms are probably conserved as well.
